# Antireflux mucosal intervention for refractory gastroesophageal reflux disease after multiple Nissen fundoplications

**DOI:** 10.1055/a-2607-8529

**Published:** 2025-06-18

**Authors:** Masachika Saino, Haruhiro Inoue, Kei Ushikubo, Kazuki Yamamoto, Yohei Nishikawa, Ippei Tanaka, Mayo Tanabe

**Affiliations:** 1 378609 Digestive Diseases Center, Showa University Koto Toyosu Hospital, Tokyo, Japan

Fundoplication procedures are widely performed for refractory gastroesophageal reflux disease (GERD), but 10%–20% of patients experience persistent or recurrent symptoms postoperatively, posing a substantial challenge 1 . Revision surgery is an option but carries a higher risk of complications, and endoscopic therapy has emerged as a minimally invasive alternative. Here, we present a case in which antireflux mucosal intervention (ARMI) was successful for a patient with refractory GERD after multiple Nissen fundoplications.

A 77-year-old man with a severe hiatal hernia underwent three Nissen fundoplications over 10 years for potassium-competitive acid blocker (P-CAB)-resistant GERD, but his symptoms persisted. Endoscopy revealed Barrett’s esophagus and a loosened fundoplication ( Fig. 1 **a** ). The acid exposure time was 0% on pH monitoring owing to continued P-CAB use, while an endoscopic pressure study integrated system (EPSIS) evaluation demonstrated a flat pattern 2 . Given the patient’s preference for endoscopic treatment and the lack of alternative therapeutic options, ARMI was planned.

**Fig. 1:**
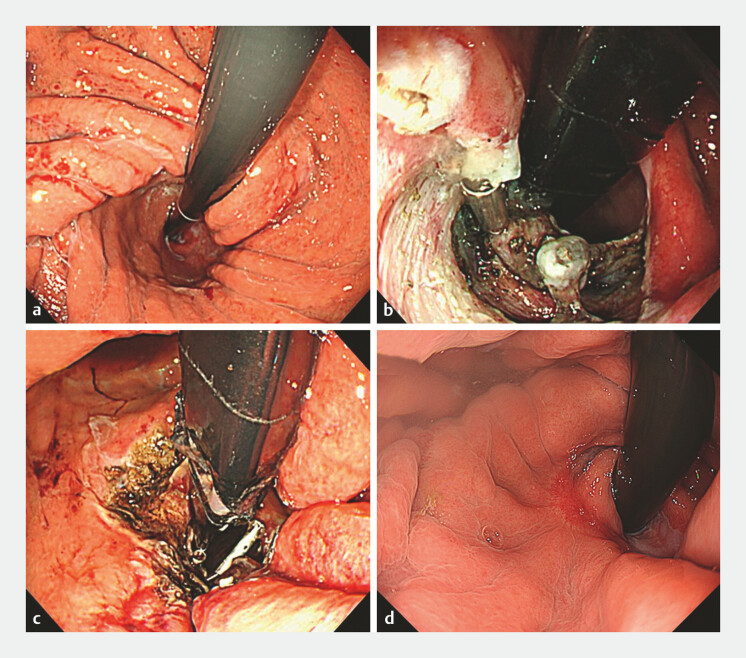
Endoscopic images showing: **a** before ARMI, the loosened fundoplication; **b** the appearance after submucosal dissection and valve formation; **c** inspection following additional mucosal ablation; **d** 3 months post-treatment, improved morphology of the esophagogastric junction.

The procedure involved submucosal dissection just below the gastroesophageal junction, followed by inversion and fixation of the dissected mucosa with clips to create a valve structure ( Fig. 1 **b** ; Video 1 ). Argon plasma coagulation was applied to the surrounding mucosa ( Fig. 1 **c** ). The postoperative course was uneventful, with no adverse events experienced.

Antireflux mucosal intervention for gastroesophageal reflux disease after multiple fundoplications using mucosal incision and ablation.Video 1

At the 3-month follow-up, the GERD Health-Related Quality of Life score had improved from 17 to 2, and P-CAB therapy was no longer required. Endoscopy confirmed reinforcement of the gastroesophageal flap valve ( Fig. 1 **d** ). Because of the improvement in symptoms, the patient refused post-treatment pH monitoring, but EPSIS re-evaluation revealed an uphill pattern 3 .

Reoperative fundoplication for refractory GERD is often challenging. Treatment decisions are particularly difficult in patients where multiple surgeries have failed to achieve symptom relief. While the efficacy of ARMI in patients with a history of surgical antireflux procedures remains uncertain, this report highlights an important case suggesting that ARMI may serve as a viable alternative for such patients.

Endoscopy_UCTN_Code_TTT_1AO_2AJ

## Notes

Endoscopy E-Videos https://eref.thieme.de/e-videos
*E-Videos* is an open access online section of the journal *Endoscopy* , reporting on interesting cases and new techniques in gastroenterological endoscopy. All papers include a high-quality video and are published with a Creative Commons CC-BY license. Endoscopy E-Videos qualify for HINARI discounts and waivers and eligibility is automatically checked during the submission process. We grant 100% waivers to articles whose corresponding authors are based in Group A countries and 50% waivers to those who are based in Group B countries as classified by Research4Life (see: https://www.research4life.org/access/eligibility/ ). This section has its own submission website at https://mc.manuscriptcentral.com/e-videos .
